# Impact of Training Data, Ground Truth and Shape Variability in the Deep Learning-Based Semantic Segmentation of HeLa Cells Observed with Electron Microscopy

**DOI:** 10.3390/jimaging9030059

**Published:** 2023-03-01

**Authors:** Cefa Karabağ, Mauricio Alberto Ortega-Ruíz, Constantino Carlos Reyes-Aldasoro

**Affiliations:** 1 giCentre, Department of Computer Science, School of Science and Technology, City, University of London, London EC1V 0HB, UK; 2 Departamento de Ingeniería, Campus Coyoacán, Universidad del Valle de México, Ciudad de México C.P. 04910, Mexico

**Keywords:** U-Net, deep learning, training, cell segmentation, HeLa cells

## Abstract

This paper investigates the impact of the amount of training data and the shape variability on the segmentation provided by the deep learning architecture U-Net. Further, the correctness of ground truth (GT) was also evaluated. The input data consisted of a three-dimensional set of images of HeLa cells observed with an electron microscope with dimensions 8192×8192×517. From there, a smaller region of interest (ROI) of 2000×2000×300 was cropped and manually delineated to obtain the ground truth necessary for a quantitative evaluation. A qualitative evaluation was performed on the 8192×8192 slices due to the lack of ground truth. Pairs of patches of data and labels for the classes nucleus, nuclear envelope, cell and background were generated to train U-Net architectures from scratch. Several training strategies were followed, and the results were compared against a traditional image processing algorithm. The correctness of GT, that is, the inclusion of one or more nuclei within the region of interest was also evaluated. The impact of the extent of training data was evaluated by comparing results from 36,000 pairs of data and label patches extracted from the odd slices in the central region, to 135,000 patches obtained from every other slice in the set. Then, 135,000 patches from several cells from the 8192×8192 slices were generated automatically using the image processing algorithm. Finally, the two sets of 135,000 pairs were combined to train once more with 270,000 pairs. As would be expected, the accuracy and Jaccard similarity index improved as the number of pairs increased for the ROI. This was also observed qualitatively for the 8192×8192 slices. When the 8192×8192 slices were segmented with U-Nets trained with 135,000 pairs, the architecture trained with automatically generated pairs provided better results than the architecture trained with the pairs from the manually segmented ground truths. This suggests that the pairs that were extracted automatically from many cells provided a better representation of the four classes of the various cells in the 8192×8192 slice than those pairs that were manually segmented from a single cell. Finally, the two sets of 135,000 pairs were combined, and the U-Net trained with these provided the best results.

## 1. Introduction

The immortal *HeLa* cell line, which originated from cervical cancer cells of the patient Henrietta Lacks, is the oldest and most commonly used human cell line [1,2]. These cells have been widely used in numerous experiments: from cancer [3,4,5,6,7] to toxoplasmosis [8,9,10,11] to AIDS [12,13,14,15] to radiation [16,17,18,19]. The story of these cells is not just a matter of scientific literature. Recently, it became the subject of books and films based on the life of Henrietta Lacks. The cells were extracted, stored and distributed without the consent or knowledge of the patient or her family, as this was not a requirement in 1951 in the United States. The remarkable and engaging story that the cells followed is narrated by Rebecca Skloot in her book *The immortal life of Henrietta Lacks* [20] and a film under the same name.

One area of interest in cancer research is the observation of the distribution, shape and morphological characteristics of the cells and cellular structures, such as the nuclear envelope (NE) and the plasma membrane [21,22,23,24]. Whilst it is possible to observe these characteristics of the cells visually, or to manually delineate the structures of interest [25,26,27,28], automatic segmentation of the cell and its structures is crucial for high throughput analysis where large amounts of data, i.e., terabytes, are acquired regularly.

Segmentation is an essential and difficult problem in image processing and machine vision for which a great number of algorithms have been proposed. The particular case of the segmentation and classification of cells and their structures remains as one important and challenging problem of interest for the clinical and programming communities [29,30]. Segmentation can be understood as a process in which an image is divided or partitioned into objects or regions, say object/background, or $object_{a}/object_{b}/\ldots background$. The partition considers that the regions should be “non-intersecting, such that each region is homogeneous and the union of no two homogeneous regions is homogeneous” [31]. When the data are medical or biomedical, the regions usually correspond to anatomical or biological structures [32] e.g., cells, nuclei, cartilages or tumors. Some authors consider that the segmentation and subsequent reconstruction of the shape of medical organs or biological structures is harder than other computer vision problems because of inherent complexity, large shape variability, and characteristic artifacts of the acquisition systems [33]. The large number of segmentation methods in the literature is an indication of the complexity and importance of the problem. At the time of writing this paper (January 2023), PubMed reported more than 50,000 entries for “segmentation” (https://pubmed.ncbi.nlm.nih.gov/?term=(“segmentation”) (URL accessed 23 February 2023)).

Some of the most common segmentation techniques are the following. Grey level thresholding allocates a single or multiple thresholds so that pixels with a grey level lower than the threshold belong to one region and the remaining pixels to another region. The thresholding methods rely on the assumption that the objects to segment are distinctive in their grey levels and use the histogram information, thus ignoring the spatial arrangement of pixels. Whilst these techniques are probably the most basic ones, they are still widely used [34,35,36]. Iterative pixel classifications, such as Markov random fields (MRFs) rely on the spatial interrelation of the pixels with its neighbors. Energy functions, compatibility measures or training of networks determine the classification. In particular, MRFs [37,38,39] have been widely used in the classification of medical images with good results. Superpixel segmentation methods [40] group pixels that share certain characteristics and whilst doing so, reduce significantly the number of elements to be segmented or classified and thus have become a standard tool of many segmentation algorithms [40,41,42]. The watershed transform [43,44] considers not only the single intensity value of a pixel, but its relation with its neighbors in a topographical way. That is, regions are considered homogeneous when they belong to the same catchment basin through which idealized “rain” would flow toward a “lake”. The watershed provides a partition for very different thresholding, and thus is a very useful step of many segmentation algorithms [45,46]. Active contours define a curve or “snake”, which iteratively evolves to detect objects in an image based on constraints within the image itself [47]. Since the curve can evolve over a large number of iterations, the segmentation is very versatile, and even complex shapes, such as the corpus callosum [48], heart ventricles [49] or megakaryocitic cells [50] can be accurately segmented.

Machine learning and deep learning techniques have grown significantly in recent years [51] and a large number of architectures have been proposed with excellent results [52,53]. Whilst deep learning removes the need to hand-craft algorithms, one of the disadvantages is the scarcity of large datasets of training data. The training data of biomedical datasets are generally generated by manual delineation by a single expert, a group of experts or through citizen-science approaches [54]. Automatic labeling for the generation of training data is an attractive alternative to the time-consuming manual interventions. In areas different from biology, the use of synthetic datasets is a useful approach to generate training data [55,56]. Similarly, in some cases, the labels of well-defined objects are simplified, i.e., in the context of navigation, moving objects, such as cars or pedestrians, are labeled as moving, and all other objects are labeled as stationary [57]. In the context of synthetic aperture radar, it is possible to fuse together the image data themselves with a separate source of information, i.e., OpenStreetMap [58] or extra images from the Earth observation mission Sentinel-2 [59], to generate labels. In cell biology, and especially as observed with electron microscopy, none of these approaches are feasible due to the inherent variability of cells and other objects, which are less characteristic than a car or a pedestrian and the lack of alternative sources of information at the required resolution.

The segmentation of cells, their structures and the characteristics obtained from the segmentation, i.e., morphology or numbers, is crucial in the diagnosis of disease [60,61,62,63] and eventually can impact the treatment selected [64]. The segmentation of NE and the plasma membrane depends on the resolution of the acquisition equipment and the contrast it provides, as well complexity of the structures themselves. At higher resolutions and in three dimensions, such as those provided by electron microscopy, the problem is challenging [65,66], and manual delineation is used [67,68], sometimes through citizen-science approaches [54]. Segmentation with traditional image processing algorithms and deep learning approaches [69] are widely used in tasks of segmentation, and have previously been compared for the segmentation of NE and plasma membranes of HeLa cells as observed with electron microscopes [70].

Previous work on Hela cells by the authors focused initially on the development of an automated segmentation of HeLa nuclear envelope, i.e., a hand-crafted traditional image processing algorithm [71], and the modeling of the segmented three-dimensional nuclear envelope against a spheroid to create a two-dimensional (2D) surface [72] of a single cell, which was manually cropped from a larger volume ( 8192×8192×517 volume elements (voxels)) into a region of interest (2000×2000×300). The algorithm was then compared against four deep learning segmentation approaches, namely, VGG16, ResNet18, Inception-ResNet-v2 and U-Net [73]. The traditional image processing algorithm provided better results than the other approaches. However, it was observed that whilst the image processing algorithm segmented only the central nucleus, the U-Net segmented all nuclei that appeared in the images. Thus, the comparison was not *like-with-like*. A parallel work on HeLa cells [74] exploited the power of a citizen-science approach to enable volunteers to manually segment the nuclear envelope of the HeLa cells. The aggregated segmentations were then used to train a U-Net and segment 20 cells from the data. The image processing algorithm was further developed in [73,75] to segment the plasma membrane in addition to the nuclear envelope. Further, whilst the previous papers had worked with a region of interest, Ref. [75] expanded the scope to a larger dataset and performed an instance segmentation of 25 cells. It is important to highlight that a limitation of the algorithm is that the cells were detected separately and then segmented in an iterative manner. An approach that could segment all the cells in one slice would be an improvement over the algorithm.

In this work, the impact that the training data can have on the outcome of a segmentation was evaluated, and a comparison of the segmentations of HeLa plasma membranes and NE with a U-Net [76] was performed. Furthermore, the possibility of generating training data through the use of the automatic segmentation with a traditional image processing algorithm was explored. In this way, the training data were expanded to include pairs from the hand-segmented regions and the automatically-segmented regions.

**Figure 1:**
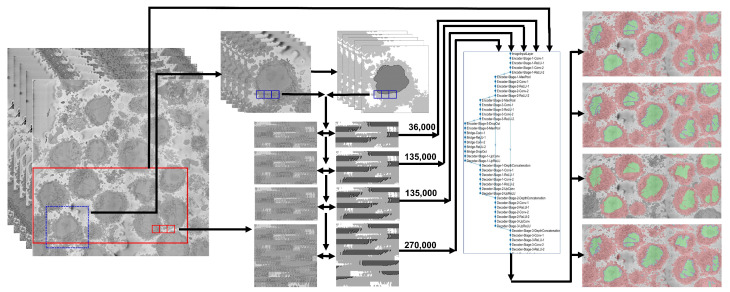
Graphical abstract of the framework analysed in this paper. The input data consist of 517 electron microscope images with dimensions 8192×8192 pixels. A region of interest (ROI) (blue box) of 300 images each 2000×2000 was cropped and manually labeled into 4 classes (nucleus, nuclear envelope, cell, and background) to generate a ground truth. Pairs of patches of data and labels were generated and used to train a U-Net architecture with different strategies: 36,000 pairs, 135,000 pairs from a single cell, 135,000 from all the dataset extracted automatically with the image processing algorithm, and finally 270,000 with 135,000 from the ROI and 135,000 from other cells. The segmentations for the ROI were evaluated quantitatively, and the accuracy and Jaccard index were calculated. For a larger subsection of the data (red box), the evaluation was qualitative due to lack of ground truth.

Specifically, a region of interest (ROI) of 2000×2000×300 volume elements (voxels) was cropped from a larger dataset of 8192×8192×517 voxels, and was manually delineated to obtain a ground truth (GT). The ROI was segmented under the following scenarios: (a) training a U-Net from scratch with 36,000 patches for data and labels from alternate slices of the central region of the cell (101:2:180) considering that there was a single cell in the ROI, (b) training a U-Net from scratch considering the existence of multiple cells in the ROI and 36,000 patches for data and labels from alternate slices of the central region of the cell (101:2:180), and (c) training a U-Net from scratch considering a multiple cells in the ROI and 135,000 patches for data and labels from alternate slices of the whole cell (1:2:300). The U-Net model trained in (c) was then used to segment 8192×8192 slices, voxels and then (d) a new U-Net was trained from scratch considering the 135,000 pairs of patches from (c) plus 135,000 new patches that were generated automatically through the segmentation of an image processing algorithm. The results for the 2000×2000×300 images of the region of interest were compared slice-per-slice against a previously published traditional image processing algorithm using accuracy and the Jaccard similarity index. The results of the 8192×8192 slices were visually assessed, as there was no ground truth available. A graphical abstract of the work is presented in Figure 1. All the programming was performed in Matlab^®^ (The Mathworks^TM^, Natick, MA, USA).

Thus, the main contributions of this work are summarized as follows:

- The impact of the amount and nature of training data on the segmentation of HeLa cells observed with an electron microscope was evaluated in quantitative and qualitative comparisons.
- A methodology to automatically generate a ground truth using a traditional image processing algorithm is proposed. This ground truth was used to generate training pairs that were later used to train a U-Net. The ground truth was obtained from several cells in several 8192×8192 slices.
- Data, code and ground truth were publicly released through Empiar, GitHub and Zenodo.

## 2. Materials and Methods

### 2.1. HeLa Cells Preparation and Acquisition

**Figure 2:**
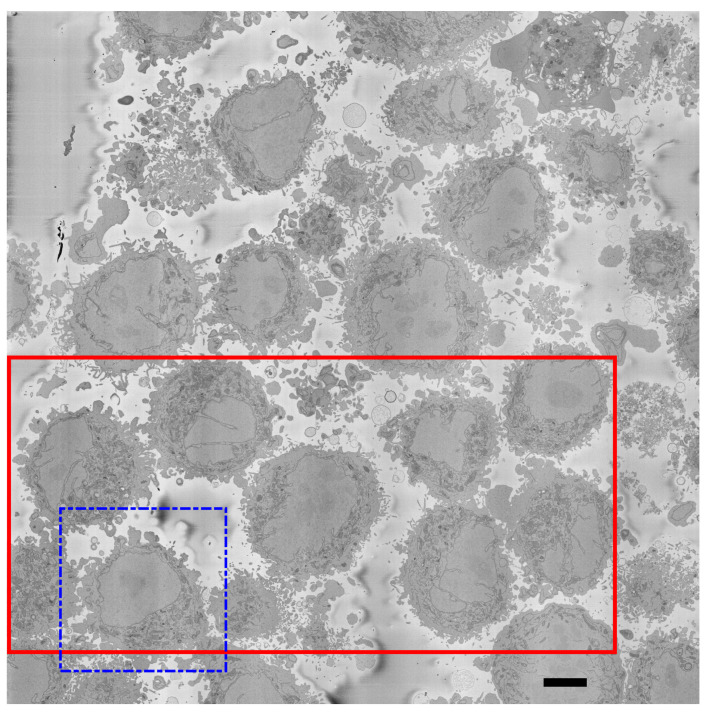
One representative slice of one of the 8192×8192×517 images. The image shows several HeLa cells which are darker than the background. It should be noticed that the background is not completely uniform in intensity but it is smooth, and changes of intensity are not as abrupt as those for the cells. The main visible characteristic of the cells is a large central region that corresponds to the nucleus, surrounded by a dark thin region corresponding to the nuclear envelope. Cells with different characteristics are visible: for some, the nucleus is relatively smooth and regular (dashed blue box; this will be referred to in the manuscript as the region of interest (ROI)) as opposed to other nuclei that present invaginations and more complex geometries. Other structures, such as mitochondria are visible, but these are outside the scope of this work. A solid red box shows a larger region of interest, which will be used for qualitative comparisons. Scale bar at the bottom right indicates 5 μm.

**Figure 3:**
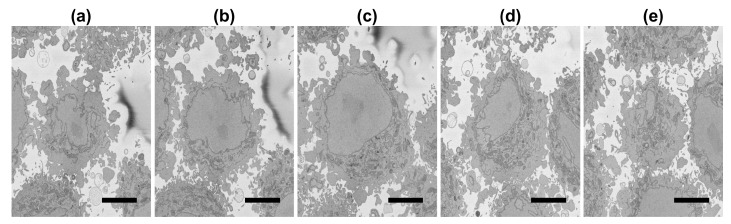
(**a**–**e**): Five representative slices of the 2000×2000×300 region of interest (ROI) with the central slice (**c**) corresponding to the blue box in Figure 2. It should be noticed how the nucleus decreases in size towards top (**d**,**e**) and bottom (**a**,**b**) of the cell. Also, the nuclei of the cells that surround the central cell are particularly visible towards the bottom of the cell (**a**) at the left side and top of the cell (**e**) at the right and bottom of the image. Scale bar at the bottom right indicates 5 μm.

Details regarding the preparation of the HeLa cells have been previously described [75]. For completeness, these are briefly described here. Cells were embedded in Durcupan and observed with an SBF scanning electron microscope following the National Center for Microscopy and Imaging Research (NCMIR) protocol [77]. The images were acquired with a SBF SEM 3View2XP microscope (Gatan, Pleasanton, CA, USA) attached to a Sigma VP SEM (Zeiss, Cambridge, UK). Voxel dimensions were 10 × 10 × 50 nm with intensity values in the range [0–255]. Five hundred and seventeen images of 8192×8192 pixels were acquired. A ROI was cropped by estimating manually the centroid of one cell at the centre of a box of 2000×2000×300 voxels. Figure 2 illustrates a representative slice of one of the 8192×8192 images with the ROI indicated by a dashed blue square and a solid red line a larger region to illustrate the qualitative evaluation. Figure 3 illustrates several slices of the 2000×2000 ROI. All the EM dataset are publicly available through EMPIAR [78] at the following URL: http://dx.doi.org/10.6019/EMPIAR-10094 (accessed on 23 February 2023).

### 2.2. Ground Truth (GT)

Ground truth for the 2000×2000×300 ROI was manually delineated slice-per-slice in Matlab. Disjoint nuclear regions were assessed by scrolling up and down to decide whether to include them as part of the nucleus. The ground truth for the ROI with (a) only the central nucleus and (b) the central nucleus and other nuclei are publicly available through Zenodo: https://doi.org/10.5281/zenodo.3874949 (accessed on 23 February 2023) for single nucleus, and https://doi.org/10.5281/zenodo.6355622 (accessed on 23 February 2023) for multinuclei.

### 2.3. Traditional Image Processing Segmentation Algorithm

**Figure 4:**
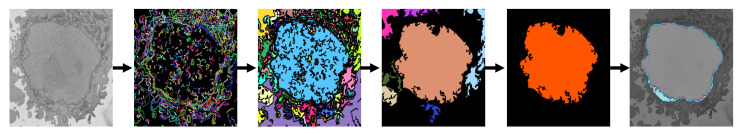
Graphical illustration of the image processing algorithm. The image is low-passed filtered to reduce noise, edges are generated and then the regions that are not covered by edges become superpixels. Morphological operations are used to discard all regions except the one that corresponds to the nucleus, which in this case is the large central one. Finally, closing operations are used to fill the small gaps and provide a smooth nuclear segmentation.

The semantic segmentations obtained with the different configurations of the U-Net were compared against a traditional image processing algorithm. Details of the algorithm have been described previously [72], but briefly, the algorithm is based on a pipeline formed of traditional image processing steps that are applied to a ROI that has been cropped from the whole dataset. The first step is low pass filtering to reduce the noise intrinsic to the acquisition of the electron microscope. Next, the areas of the image where the intensity changed abruptly were detected with the canny edge detection algorithm [79]. The nuclear envelope is not the only region of abrupt changes and thus, a large number of edges were detected. However, the edges were not used directly; rather, these were used to generate a series of superpixels which corresponded to all the areas of the image where edges did not exist. Again, a large number of superpixels were generated. Next, a series of morphological operations were applied. Specifically, small regions and those in contact with the edge of the image were discarded. Further, the central region of the image was selected, and then closing operations were used to smooth the final central region. This process was repeated on a slice-by-slice basis, starting from the central slice and propagating the results up and down the 3D stack. This process provided the combination of adjacent slices to select disjoint regions that belong to the nucleus. A simplified pipeline is illustrated graphically in Figure 4. This algorithm obtained accurate semantic segmentations, which outperformed other deep learning approaches [73]. However, in the case of U-Net, it was noticed that the inferior accuracy and Jaccard index were due to the discrepancy between the segmentation, which detected several nuclei, and the ground truth, which considered only the central nucleus. The Matlab code is available through GitHub: (https://github.com/reyesaldasoro/HeLa-Cell-Segmentation (accessed 23 February 2023)).

### 2.4. U-Net Architecture

**Figure 5:**
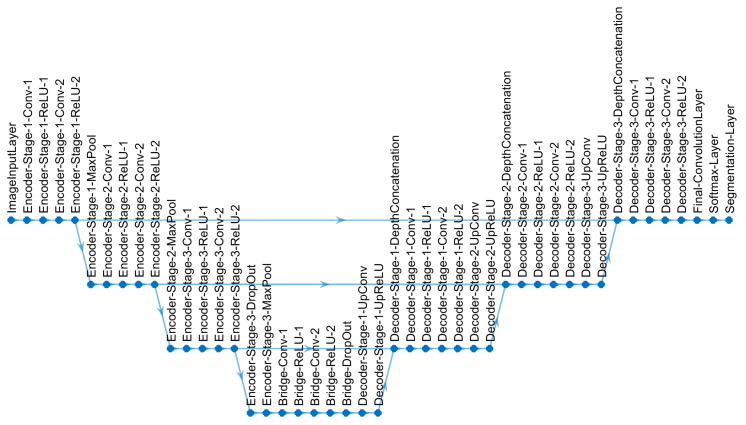
U-Net architecture used for semantic segmentation in this work. Each level consists of a series of operations: convolutions (abbreviated as Conv), non-linear operations (rectified linear unit or ReLU) and subsampling (MaxPool). The data are first reduced in resolution (going down in the diagram) and then expanded (going up) to return to the same level of the input image.

The U-Net architecture [76] is a convolutional neural network architecture, which has been widely used for the segmentation of medical images. To cite just a few, U-Nets have been used in the segmentation of osteosarcoma in computed tomography scans [80], lung tumors in CT scans [81], lesions of the breast in ultrasound images [82], tumors of the bladder in cystoscopic images [83], breast and fibroglandular tissue in magnetic resonance [84] and nuclei in hematoxylin and eosin-stained slices [85]. The essence of the U-Net architecture is a combination of downsampling steps (also known as the contracting path) obtained by convolutions and downsampling, which are followed by upsampling steps (also known as the expansive path) (Figure 5). The contracting path is formed by a series of operations: 3×3 convolutions (i.e., the multiplication of each of the pixels of a small 3×3 grid of the image against a filter or kernel and then added together, then the grid is shifted, and the multiplication is repeated until all pixels of the image are covered); by using several kernels, many features can be obtained and the dimensions of the data increase; rectified linear units or ReLUs (i.e., an operation that converts to zero all negative values and maintains the positive values; this is used as an activation function); and a max-pooling operation, (i.e., the maximum value of the output is selected, by selecting one value out of several, the data is being reduced in dimensions). The expansive path follows the reverse process; the features are reduced, and the dimensions are expanded to return to the original size of the input image. There is a final layer in which classes are allocated to each pixel according to the feature vectors that have been created in the process. The number of times that the data are down- or up-sampled, or going up or down, determines the depth of the architecture, and due to the rough shape of a letter “U”, the architecture is called a U-Net.

For this work, a semantic segmentation framework with a U-Net architecture was implemented in Matlab. The U-Net hyperparameters are the following:

- Adam optimizer [86],
- four levels of depth, with 46 layers as shown in Figure 5,
- size of the training patches = 128×128 pixels,
- number of classes = 4 (background, cell, nuclear envelope, nucleus),
- number of epochs for training = 15,
- Initial Learn Rate = 1 $\times \;10^{-3}$,
- Mini Batch Size = 64.

The whole framework is illustrated in Figure 1. The hyper-parameters of the architecture followed previous work mentioned in the introduction in order to compare the findings of this work with previous results.

### 2.5. U-Net Training Data, Segmentation and Post-Processing

**Figure 6:**
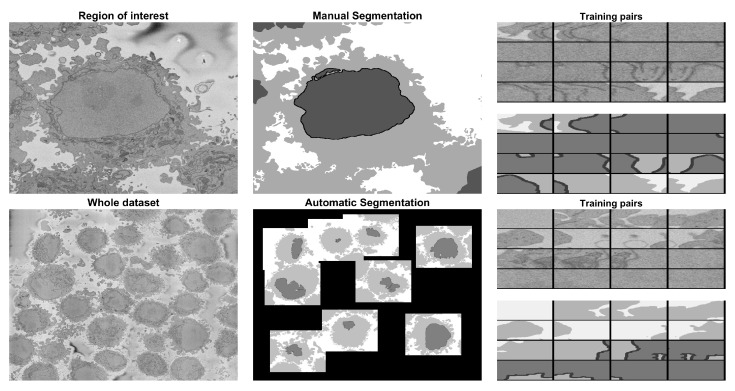
Illustration of different approaches to generate training data. The first approach shown in the top row required the manual segmentation of a 2000×2000×300 region of interest. The region was manually cropped, and then each slice was manually segmented. The image and the ground truth were used to generate pairs of patches of data and labels used in strategies 1, 2 and 3. The second approach illustrated in the bottom row applied an image processing algorithm without manual intervention. A series of cells were detected in an image. Then, in a sequential manner, each of these was automatically cropped and segmented, and the image and ground truth were used to generate pairs of patches of data and labels used in strategy 4. The number of pairs is described in Section 2.5.

The U-Net architecture can be trained end-to-end from pairs or patches of images and their corresponding classes. Once trained, the U-Net can be used to segment the input images, which for this work consisted of the 300 images of the ROI (i.e., 2000×2000) and the 517 original images (i.e., 8192×8192). To train the network for this work, the following strategies were evaluated. Details of the strategies are illustrated in Figure 6.

1. **A total of 36,000 pairs from manually delineated GT from a single cell, evaluated with a single cell in the GT**. Pairs of patches of images and labels of size 128×128 with 50% overlap were generated from 40 alternate slices of the central region of the cell (101:2:180). For one 2000×2000 image, there were 30×30 patches and thus 30×30×40 corresponded to 36,000 patches. Alternate slices were selected to exploit the similarity between neighboring slices. In this case, the ground truth included only the nucleus of the central cell visible in the ROI.
2. **A total of 36,000 pairs from manually delineated GT from a single cell, evaluated with multiple cells in the GT**. The same strategy was followed to generate 36,000 patches and labels from alternate slices of the central region of the cell (101:2:180), however, in this case, the ground truth included the nuclei of all cells visible in the ROI.
3. **A total of 135,000 pairs from manually delineated GT from a single cell, evaluated with multiple cells in the GT**. The pairs of patches of labels and data were extended to cover every other slice of the whole region of interest (1:2:300). The size was again 128×128 with 50% overlap; therefore, in this case, there were 150 slices and 900 patches per slice, which provided 135,000 pairs of patches for data and labels.
4. **A total of 135,000 pairs from automatically generated GT from multiple cells, evaluated visually**. The training was extended beyond the 2000×2000 region of interest by performing an automatic segmentation of the 8192×8192 slices. This segmentation became a novel ground truth that was used to generate the same amount of pairs and in the previous strategy. The segmentation was performed with a traditional image processing segmentation algorithm [72] previously described. Fifteen non-contiguous slices were selected in the central region of the dataset (230:10:370). In each slice, the background was automatically segmented; distance transform was calculated to locate regions furthest from background, which corresponded to the cells. The 10 most salient cells were selected in each slice. A 2000×2000 region was cropped, automatically segmented, and patches of 128×128 with 50% overlap were generated. This generated 900 patches per cell, thus 900×10×15 = 135,000. This training strategy was designed for the segmentation of the 8192×8192 slices to compare the impact of segmenting with a U-Net trained on a single cell (even with a significant number of pairs) or with pairs from more than one cell.
5. **A total of 270,000 pairs from manual (135,000) and automatic (135,000) GTs, evaluated visually**. Finally, the patches generated in the two previous strategies, that is, the 135,000 from the single cell and the 135,000 from the whole dataset were combined for a total of 270,000.

**Figure 7:**
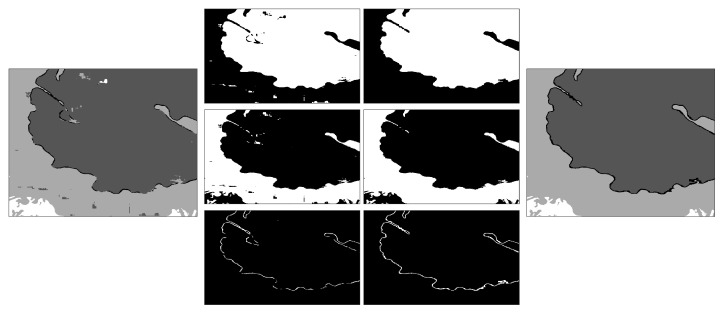
Illustration of the post-processing. The classes corresponding to a small section of one slice of the dataset are shown on the left. On the two central columns, the pre-processing and post-processing are shown per class, from top to bottom, nucleus, cell and nuclear envelope. On the right is the final post-processed region. It can be seen how small misclassifications over the nucleus and cell have been smoothed, and the nuclear envelope improved its continuity.

A simple post-processing step was applied. Each class was processed separately, and traditional image processing steps were applied: filling holes, closing with a disk structural element of 3-pixel radius, and removal of small regions (area <3200 pixels, equivalent to 0.08% of the area of the image). These steps removed small specs of larger uniform areas for the nucleus, cell and background. The nuclear envelope had an extra step: the nucleus and cell were dilated, and the overap between these two classes was added to the nuclear envelope. This removed the small discontinuities of the region. The last step aggregated the individual post-processed classes. Figure 7 illustrates the post-processing per class and the final outcome. The number of pixels modified by the post-processing is less than 1% of the data. All the Matlab code is available through GitHub: (https://github.com/reyesaldasoro/HeLa_Segmentation_UNET2, accessed on 23 February 2023).

### 2.6. Quantitative Comparisons

The segmentations assigned a class (background, nucleus, nuclear envelope, rest of the cell) to each pixel of the images. Accuracy and Jaccard similarity index (JI) [87] were calculated on a pixel-by-pixel basis for the 2000×2000×300 region of interest for which ground truth was available. For the 81,920×8192 slices, the results were visually assessed. Accuracy was calculated as (TP+TN)/(TP+TN+FP+FN) and JI, or intersection over union of nuclear area corresponded to (TP)/(TP+FP+FN), where TP corresponds to true positives, TN to true negatives, FP to false positives and FN to false negatives.

### 2.7. Hardware Details

All the processing was performed in Matlab^®^ (The Mathworks${}^{\mathrm{TM}}$, Natick, MA, USA) and executed in a Dell Alienware m15 R3 Laptop with Intel^®^ Core${}^{\mathrm{TM}}$ i9-10980HK CPU @ 2.40 GHz, 32 GB RAM, an NVIDIA^®^ GeForce RTX 2070 Super GPU Card with 8 GB RAM.

## 3. Results and Discussion

### 3.1. Results on the 2000×2000×300 Region of Interest

**Figure 8:**
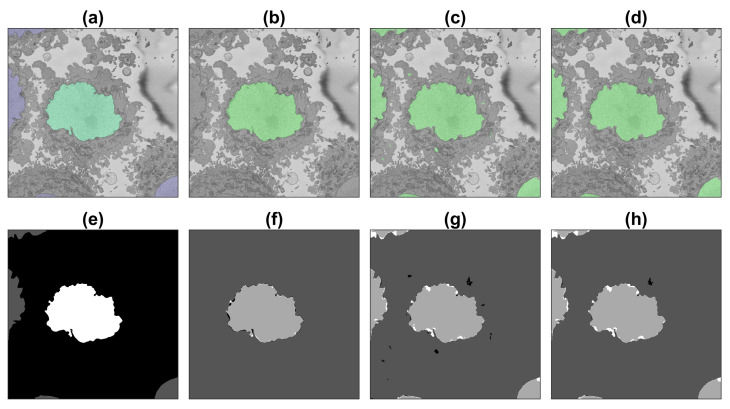
Illustration of the semantic segmentation of one slice (71/300) of the ROI with the image processing algorithm and U-Net (36,000 patches). Top row (**a**–**d**) show slice with results overlaid, bottom row (**e**–**h**) shows ground truth (GT) and results as classes. (**a**,**e**) GT, central nucleus in green/white whilst other nuclei in purple/grey and background in greyscale/black. (**b**,**f**) IP segmentation. (**c**,**g**) U-Net segmentation. (**d**,**h**) U-Net segmentation after post-processing. In (**f**–**h**) true positives = light grey, true negatives = darker grey, false positives = black, false negatives = white.

**Figure 9:**
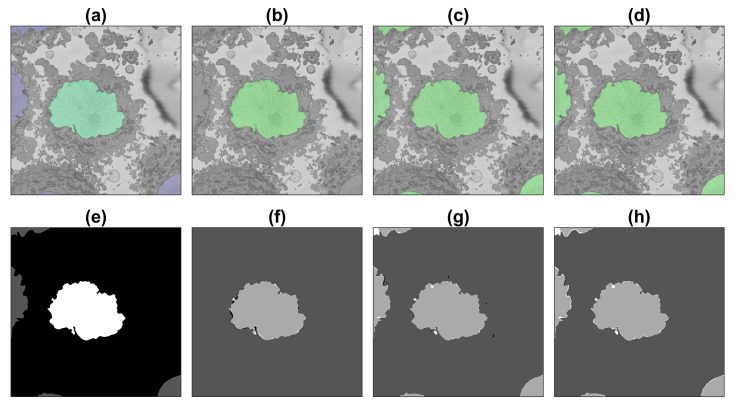
Illustration of the semantic segmentation of one slice (71/300) of the region of interest (ROI) with the image processing (IP) algorithm and U-Net trained with 135,000 patches. For a description of (**a**–**h**), see Figure 8.

**Figure 10:**
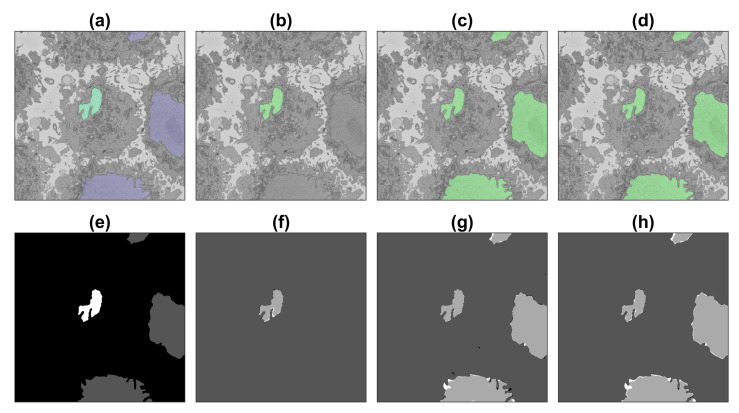
Illustration of the semantic segmentation of one slice (251/300) of the region of interest (ROI) with the image processing (IP) algorithm and U-Net trained with 135,000 patches. For a description, see Figure 8.

Segmentation of all slices of the 2000×2000×300 region of interest with the first three training strategies (i.e., excluding the patches obtained with the image processing algorithm) and calculation of accuracy and Jaccard index was performed as described in previous sections. Figure 8, Figure 9 and Figure 10 illustrate the results obtained with the image processing algorithm and the U-Net with different training strategies.

**Figure 11:**
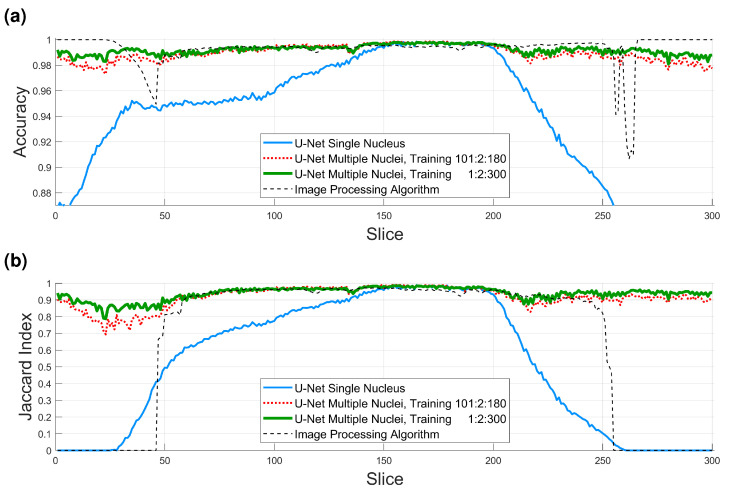
Numerical comparison of the four algorithms (U-Net single nucleus 36,000 patches, U-Net multiple nuclei 36,000 patches, U-Net multiple nuclei, 135,000 patches, image processing (IP) algorithm). (**a**) Accuracy. Average values for all slices (0.9346, 0.9895, 0.9922, and 0.9926), for slices 150:200 (0.9966, 0.9974, 0.9971, and 0.9945). (**b**) Jaccard similarity index (JI). Average values for all slices (0.5138, 0.9158, 0.9378, and 0.6436), for slices 150:200 (0.9712, 0.9778, 0.9760, and 0.9564).

Accuracy and Jaccard index results are shown in Figure 11 and in Table 1. Several points are worth mentioning. When the U-Net was trained and measured with GT with a single nucleus (solid blue line in Figure 11), the results were good between slices 150 and 200 of the ROI, as these slices contained only one nucleus. Toward the top and bottom of the ROI, the accuracy and JI dropped as other nuclei appeared in the images, and these were segmented, but were not part of the GT, and consequently, the numbers dropped. The results of the image processing algorithm (thin dashed black line in Figure 11) were very good, especially toward the central region of the cell, where the nucleus is largest. These results considered only the central cell. Toward the top and bottom of the cell, where the nucleus is smaller and the shape less regular, the accuracy remained high due to the large number of TN, but the JI dropped in a similar way to the U-Net with a single nucleus and was zero in the extremes due to the absence of TP.

For the U-Net trained with multiple nuclei and 36,000 patches (dotted red line in Figure 11), the results were significantly higher than the single nucleus U-Net, close to the image processing algorithm in central slices and better in the top/bottom, as both metrics remained high. However, for the U-Net with 135,000 patches (thick green solid line in Figure 11), the results were higher still, especially in the regions outside the slices from which the 36,000 patches were generated (i.e., 1:2:99 and 181:2:300). The average accuracy for all slices for the four algorithms was as follows: U-Net single nucleus 36,000 patches, **0.9346**, U-Net multiple nuclei 36,000 patches, **0.9895**, U-Net multiple nuclei, 135,000 patches, **0.9922**, and image processing algorithm **0.9926**. In general, all these results are very high for all cases where the appropriate GT is considered, but the high values also indicate a large number of TN, especially on the top and bottom slices. For the central slices (150:200), where there is a single cell and results are best, the values are all above 99% (same order **0.9966, 0.9974, 0.9971, 0.9945**); thus, the errors are due to small variations on the edge of the nucleus or the fact that the manual delineation can also include small errors. The values for the Jaccard index are more interesting, with the average values for all slices of (**0.5138, 0.9158, 0.9378, 0.6436**), it is clear that the multinuclei U-Nets have a significant improvement, as they consider better the top and bottom slices. For the central slices (150:200), the values are pretty similar for the three U-Nets and slightly lower for the image processing (**0.9712, 0.9778, 0.9760, 0.9564**), as a small dip is visible around slice 180. A final comparison in the Jaccard, between slices 60:150, where the curves are very similar, shows the following values (**0.8047, 0.9579, 0.9592, 0.9565**), that is, nearly identical averages for all methods, except the initial U-Net. These results confirm that the larger the training data, the better the U-Net can learn the characteristics of the cells. It is important to highlight that the results are not completely like-with-like, as the GT is not the same for the single nucleus and the multinuclei approaches.

### 3.2. Results on the 8192×8192 Slices

**Figure 12:**
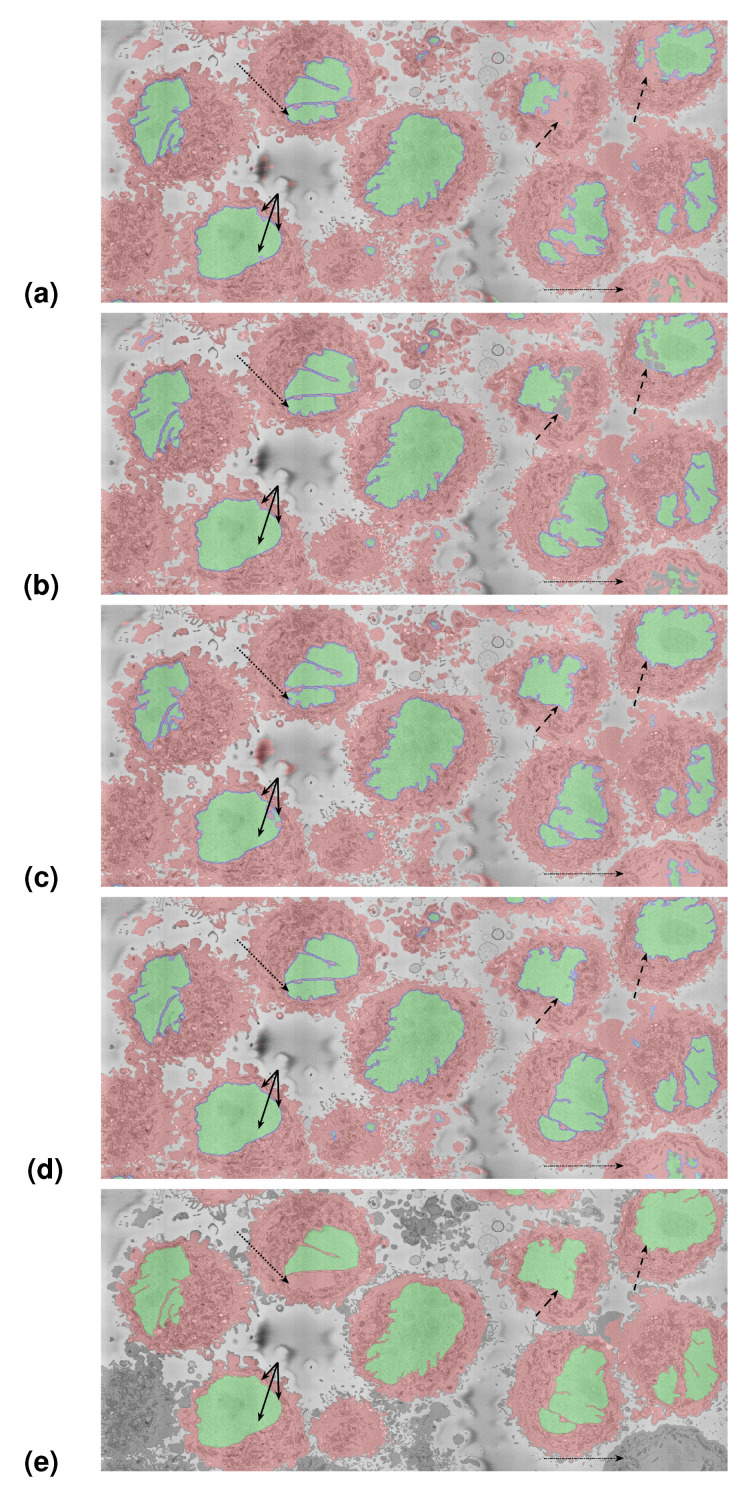
Segmentation of the section of interest shown in Figure 2 (red box). (**a**) U-Net trained with 36,000 patches from one cell (bottom left). (**b**) U-Net trained with 135,000 patches from one cell. (**c**) U-Net trained with 135,000 patches automatically generated with the image processing algorithm. (**d**) U-Net trained with 270,000 patches; the combination of the 135,000 of previous approaches. (**e**) Image processing (IP) algorithm. Cells are shown with red shading, nuclei are shown with a green shade, and for the U-Net results, the nuclear envelope is shown with a blue shade. It can be noticed that the segmentation of the single cell from which patches were extracted (bottom left, solid arrows) is good in all cases, with slight errors in (**c**), which is natural, as the training for this strategy originated from other cells. On the other hand, the segmentation of the top right cells (dashed arrows) is much better in (**c**) than (**a**) or (**b**), which shows the improvement provided by training in more than one cell. Whilst the image processing algorithm provides very good results, it proceeds iteratively, and unlike the U-Net, it will not process all the cells as those in the edges (dashed-dot arrow in the bottom right), and in some cases, it may miss a section of the nucleus (dashed arrow in the top left).

As a further segmentation test, the U-Nets trained on multiple nuclei were used to segment the 8192×8192 slices. The results were evaluated visually due to the lack of ground truth. Figure 12 shows the results of the U-Nets and the image processing algorithm. Figure 12a shows the segmentation obtained with the U-Net trained with 36,000 pairs, Figure 12b shows the segmentation with 135,000 pairs from the single cell from the ROI, Figure 12c shows the segmentation with 135,000 pairs automatically generated with the image processing algorithm, Figure 12d shows the segmentation with 270,000 pairs, and Figure 12e shows the segmentation with the image processing algorithm.

The first observation is that as the training sets grow, the U-Nets provide better results than those trained with smaller sets. This improvement can be observed in several locations: The cells on the top right show a discontinuous nucleus in (a), then a nucleus with holes in (b). These less than satisfactory results on (a,b) may be the outcome of training the U-Nets on a single cell, whose nucleus is considerably smoother than those of the surrounding cells. The second observation is the improvement that is provided with the pairs generated automatically with the image processing algorithm (c). The nuclei on the cells on the top right are much better segmented (dashed arrows). The cell from the ROI on the other hand (bottom left, solid arrows) now presents some small holes inside the nucleus. This is not surprising, as the training data are not from this cell in particular. The results shown in (d) combined the pairs from the ROI and the automatically generated and show improvement in the cell from the ROI and other cells. The sharper delineation of the invaginations should be noticed. These can be compared with the image processing algorithm (e), which provided results equivalent to the segmentation with the network trained with 270,000 pairs.

Training with a larger number of training patches improved the results, but the key element is not necessarily the number of training pairs, but the fact that the training pairs were extracted *from different cells*. This is important, as just a larger number of samples or an enlargement with augmentation methods will not necessarily improve results unless the samples are representative of the data to be segmented.

The image processing algorithm was run on an instance segmentation, that is, individual cells in 2000×2000 regions of interest were automatically cropped and segmented. Only a reduced number of cells per slice were selected, which can be noticed in that some cells (bottom right and left) are not segmented and appear grey. It can also be noticed that some cellular regions are not segmented, especially those that are between cells. However, the shapes of the nuclei appear well segmented, with the exception of the second cell from the left on the top, for which a section of the nuclei was not detected.

## 4. Conclusions

The quantitative and qualitative comparisons of different semantic segmentation approaches of HeLa cells, their nucleus, nuclear envelope, cell and background, are presented in this work. Whilst, as would be expected, the fact that a larger amount of training data provides more accurate segmentation was sustained; it was further observed that the variability of the cells, even when it is a single line of cells, precludes the generalization that training data can be extended from one cell to another without degradation of the results. The addition of training data from a variety of cells improved considerably the segmentation results, and the combination of two sources further improved the results. Moreover, the consideration of the correct ground truth can be extremely important in the assessment of results of different approaches. An immediate observation is that this research is limited to a single dataset, and even when considerably large, with numerous cells, it is just one dataset. The cells themselves show significant variability as can be seen in Figure 12. It is possible that at lower resolutions, this variability is less significant, and thus, the data used to train a U-Net may have lower impact in the results.

In the context of this work, the main advantage of the U-Net architecture (against an image processing algorithm) is the capability to segment the images at full resolution (8192×8192), without the need to crop into regions of interest select individual cells. Whilst it was not developed in this work, a semantic segmentation of the cells could be integrated as a post-processing step of the U-Net framework. The first disadvantage of the U-Net algorithm is the requirement of training data, which many times requires manual labeling. Further, the amount and diversity of the training data can have a strong impact on the segmentation results. However, as it was demonstrated here, the negative impact can be reduced by increasing the training data with pairs generated automatically by the image processing algorithm.

The main advantage of the image processing algorithm is that it is an automatic process when a region of interest of 2000×2000×300 voxels with a single cell in the center is provided as input data. There is no need for training data. To process images at full resolution, the algorithm can detect cells and crop automatically, but in some cases, the region of interest may contain more than one cell or contain only part of the cell, especially when close to the edges of the dataset. Whilst a manual interaction is required to determine the number of cells to be processed, the segmentation results are instance based, as the cells are identified as individual cells. The main disadvantage of the algorithm is that it cannot process a dataset with multiple cells, whether this is a crop or a full resolution set. When processing the full dataset, the algorithm requires cells to be cropped into individual volumes, where it is assumed the cell will be centered. The algorithm will only segment one cell per volume.

Future work can be divided in five parallel lines. First, the proposed U-Net framework can be developed to consider the identification of individual cells for an instance-based segmentation. Given that the framework identifies nuclei, it would not be difficult to identify the number of these, which will in turn identify the number of cells present. The difficulty would be in the separation of individual cells, as was performed by traditional image processing algorithms on a cell-by-cell basis [75]. The algorithm first identified cells, cropped regions of interest per cell and segmented one cell per cropped region of interest.

Second, the identification of other sub-cellular structures, such as mitochondria and the Golgi apparatus would be of great interest and could be incorporated into the framework. For the U-Net, a number of training pairs of images and patches, which include the organelles, should be generated. An image processing approach can exploit the visual characteristics, such as the round structure crossed by lines shown by the mitochondria.

Third, the image processing algorithm and U-Net architecture were applied directly, following the use of these in previous publications. Optimization of both approaches could be investigated. For the image processing algorithm, a sensitivity analysis of key parameters, such as structural elements, could be performed. For the U-Net, different optimization techniques could be explored. For example, broad ablation studies could be performed; exploration of the number of layers, epochs, optimizer, loss function, residual connections or decoder attention [88,89,90]. The modification or addition of layers is also a possibility, for instance, interpolation and convolution instead of transposed convolution [91], adding deconvolution and upsampling layers in the splicing process [92], or fuzzy layers in addition to the conventional layers [93].

Fourth, a limitation of the current segmentation is that it was performed on a slice-by-slice basis, without fully exploiting the three-dimensional nature of the data. The image processing algorithm does exploit the 3D by comparing the results of neighboring slices and thus propagating the results. Another option could be to perform a rotation of axes so that segmentations in other planes can be performed and the results combined. This was previously proposed as a “tri-axis prediction” [70].

Fifth, it was previously mentioned that data augmentation methodologies may not necessarily improve the training if the pairs obtained from one cell are not representative of other cells. However, a thorough investigation of different augmentations: crop and resize, rotation, cutout, flips, Gaussian noise and blur, Sobel filter [94], elastic or warping deformations [95], or deep learning methods, e.g., generative adversarial networks [96] could be considered.

## Figures and Tables

**Table 1:** Numerical results of the accuracy and Jaccard similarity index obtained in one cell for which ground truth was available. It is important to highlight that “single nucleus” and “multiple nuclei” refer to the evaluation, not the training.

	U-Net Single Nucleus 36,000 Strategy 1	U-Net Multiple Nuclei 36,000 Strategy 2	U-Net Multiple Nuclei 135,000 Strategy 3	Image Processing Algorithm
Accuracy 1:300	0.9346	0.9895	0.9922	0.9926
Accuracy 150:200	0.9966	0.9974	0.9971	0.9945
Jaccard 1:300	0.5138	0.9158	0.9378	0.6436
Jaccard 150:200	0.9712	0.9778	0.9760	0.9564
Jaccard 60:150	0.8047	0.9579	0.9592	0.9565

## Data Availability

Electron microscopy datasets are available at http://dx.doi.org/10.6019/EMPIAR-10094, ground truths is available at https://doi.org/10.5281/zenodo.3874949, https://doi.org/10.5281/zenodo.6355622. Code is available at https://github.com/reyesaldasoro/Hela-Cell-Segmentation, https://github.com/reyesaldasoro/HeLa_Segmentation_UNET2, all accessed on 24 October 2022. The HeLa cell line was obtained from The Francis Crick Institute and prepared by Christopher J. Peddie.
